# Traditional Chinese medicine interventions for post-stroke cognitive impairment: an evidence mapping

**DOI:** 10.3389/fneur.2026.1818770

**Published:** 2026-06-18

**Authors:** Qi Wang, Qingchen Lin, Yasuo Ding, Yuqin Wu

**Affiliations:** 1 Cerebrovascular Disease Center, Taizhou People's Hospital Affiliated to Nanjing Medical University, Taizhou, Jiangsu, China; 2 Department of Neurology, Taizhou People's Hospital Affiliated to Nanjing Medical University, Taizhou, Jiangsu, China

**Keywords:** cognitive function, cognitive impairment, evidence mapping, stroke, traditional Chinese medicine

## Abstract

**Background:**

Post-stroke cognitive impairment (PSCI) is a major rehabilitation concern. Traditional Chinese medicine (TCM) shows promise, yet a comprehensive, evidence-based evaluation of existing systematic reviews is lacking.

**Objective:**

This study aimed to map and evaluate the evidence on TCM interventions for PSCI, identifying strengths and gaps to inform clinical and research guidance.

**Methods:**

We searched PubMed, the Cochrane Library, Embase (via Ovid), and Web of Science, as well as Chinese databases including CNKI, Wanfang Database, the Chinese Medicine Database, and VIP Database (from their inception to December 2024) for systematic reviews or meta-analyses of TCM for PSCI. Two reviewers assessed methodological quality using A Measurement Tool to Assess Systematic Reviews 2 (AMSTAR 2). The results were synthesized using the population-intervention-comparison-outcome (PICO) framework and visualized as bubble plots in R software.

**Results:**

A total of 36 systematic reviews (all from China) were included, covering acupuncture, herbal medicine, moxibustion, auricular stimulation, and TCM exercises. AMSTAR 2 rated 33 as “critically low” and 3 as “low.” TCM demonstrated multidimensional benefits: cognitive function (36/36 beneficial), activities of daily living (26/27 beneficial), neurological function (5/5 beneficial), and overall effect (33 beneficial, 1 probably beneficial).

**Conclusion:**

TCM interventions may offer multidimensional benefits for PSCI, but the evidence is severely limited by critically low methodological quality. These findings should be considered hypothesis-generating. High-quality, prospectively registered systematic reviews and rigorous primary studies are urgently needed.

**Systematic review registration:**

CRD420251178410 (PROSPERO).

## 1 Introduction

Stroke, also known as cerebrovascular accident, is an acute cerebrovascular disease characterized by brain tissue injury caused by sudden rupture of cerebral blood vessels or obstruction of cerebral blood flow (1). Stroke remains a leading cause of death and long-term disability worldwide, and the global burden of stroke-related neurological deficits continues to rise (2). Post-stroke cognitive impairment (PSCI) refers to cognitive impairment occurring after a cerebrovascular event (3). PSCI is a common complication after stroke, with an estimated prevalence of 50–70%, and effective treatment is required to improve prognosis (3). PSCI encompasses mild cognitive impairment and dementia. It not only reduces patients’ abilities in daily living and self-care, but also hinders rehabilitation and exercise participation, thereby increasing the economic and psychological burden on families (4). Moreover, cognitive impairment increases the risk of stroke recurrence and mortality, substantially compromising quality of life and survival.

Despite advances in acute stroke management, no specific, highly effective therapy for PSCI has been established. In conventional medicine, treatment often focuses on controlling risk factors and improving cerebral circulation (5). Traditional pharmacotherapies (e.g., cholinesterase inhibitors) show limited efficacy and are frequently accompanied by adverse effects such as gastrointestinal discomfort and cardiovascular risks (5). Non-pharmacological interventions, such as cognitive rehabilitation, yield inconsistent effects and face challenges with dissemination and patient adherence (6). The complex pathophysiology of PSCI, which involves disruption of large-scale brain networks, neuroinflammation, and impaired neuroplasticity following ischemic injury, underscores the need for multifaceted therapeutic strategies (7). Therefore, exploring alternative approaches for PSCI is of important clinical value.

Traditional Chinese medicine (TCM) has a long history and a rich theoretical foundation. In TCM theory, PSCI is generally categorized as “stroke” complicated by conditions akin to “forgetfulness” and “dullness,” often attributed to deficiencies of qi and blood in the heart and spleen, insufficiency of kidney essence, liver qi stagnation, and internal obstruction by phlegm and blood stasis (8). Accordingly, TCM syndrome differentiation and treatment commonly emphasize invigorating blood circulation, resolving phlegm, and tonifying deficiency (9). Interventions, including Chinese patent medicines and acupuncture, have been widely applied for PSCI (10). Systematic reviews and meta-analyses have synthesized related evidence (11, 12), yet an overarching, field-level assessment of this evidence remains lacking.

Evidence mapping is an emerging evidence-synthesis approach that systematically collects existing evidence in a research area, performs integrative analyses and scientific appraisal, and presents the current status, problems, development directions, and evidence gaps in a concise and visually intuitive manner (13). Previous evidence mapping studies have applied this methodology to evaluate TCM interventions in stroke rehabilitation, demonstrating its utility in identifying evidence clusters and gaps across cognitive and motor outcomes (14). Compared with traditional systematic reviews, evidence maps provide a more macroscopic, multi-level perspective, with strong integrative features, both systematic and visual, thereby supporting decision-making. Given that TCM interventions for PSCI constitute an active research area, this study aims to apply evidence mapping to systematically analyze the evidence base and to inform future research directions for TCM treatment of PSCI.

## 2 Materials and methods

### 2.1 Study design

This evidence mapping aimed to systematically synthesize and evaluate existing systematic reviews (SRs) and meta-analyses (MAs) of TCM interventions for PSCI. The study followed the guidance of the Global Evidence Mapping (GEM) Initiative and adopted the Preferred Reporting Items for Systematic Reviews and Meta-Analyses (PRISMA) framework. The detailed protocol was registered on the International Prospective Register of Systematic Reviews (PROSPERO) (registration number: CRD420251178410).

### 2.2 Search strategy

We electronically searched CNKI, Wanfang Data, traditional Chinese medicine literature (SinoMed), VIP, PubMed, the Cochrane Library, Embase (via Ovid), and Web of Science from inception to December 31, 2024, limited to Chinese or English. The Chinese search strategy was: SU = (“Post-Stroke Cognitive Impairment” OR “Post-Stroke Cognitive Disorder” OR “PSCI” OR “Post-Stroke Cognitive Dysfunction”) AND SU = (“Traditional Chinese Medicine” OR “TCM” OR “Acupuncture” OR “ Chinese Herbal Medicine” OR “Chinese Herbal Formulas” OR “Moxibustion” OR “Auricular Therapy” OR “Auricular Stimulation” OR “TCM Exercise” OR “Baduanjin” OR “Tai Chi”) AND SU = (“Systematic Review” OR “Meta-analysis” OR “ Evidence-based Evaluation”).

The English search strategy (e.g., in PubMed) was: (“post-stroke cognitive impairment” OR “PSCI” OR “vascular cognitive impairment after stroke” OR “cognitive impairment post stroke”) AND (““OR “TCM” OR “acupuncture” OR “herbal medicine” OR “Chinese herbal formula” OR “moxibustion” OR “auricular therapy” OR “TCM exercise” OR “Baduanjin” OR “Tai Chi”) AND (“systematic review” OR “meta-analysis” OR “evidence-based review”).

The search strategy was adapted to the syntax and indexing system of each database. In PubMed, MeSH terms (e.g., “Post-Stroke Cognitive Impairment”) were combined with free-text words; in Embase, EMTREE terms were used. For Chinese databases (CNKI, Wanfang, VIP, SinoMed), subject-based search (SU) with the same keyword combinations was applied, as illustrated in the example string above. All searches were performed on December 31, 2024.

Grey literature was partially covered through the Chinese databases, which include dissertations and theses; two master’s theses meeting our inclusion criteria were identified and included. We did not systematically search conference abstracts or preprints because our study focused exclusively on published systematic reviews and meta-analyses. This limitation is acknowledged in the Discussion.

To identify ongoing or unpublished systematic reviews and meta-analyses, we searched the following trial registries from inception to December 31, 2024: PROSPERO,^1^ ClinicalTrials.gov,^2^ and the Chinese Clinical Trial Registry (ChiCTR).^3^ The search terms used were “post-stroke cognitive impairment” AND “traditional Chinese medicine” (or their Chinese equivalents for ChiCTR). No completed systematic reviews or meta-analyses meeting our inclusion criteria were identified; the search results consisted of ongoing registered protocols, which are listed in the supplementary materials.

### 2.3 Inclusion criteria

*Participants:* There is currently no universally accepted diagnostic gold standard for PSCI. Eligible studies included participants with clearly defined diagnostic criteria for acute ischemic stroke (AIS), with explicit conventional or TCM diagnostic standards meeting at least one Chinese or international standard, and with cognitive impairment occurring within 6 months after the stroke event. No restrictions were applied regarding sex, race or ethnicity, age, geographic region, or educational background.

*Interventions:* Any TCM therapy, such as acupuncture, Chinese patent medicines, herbal decoctions, moxibustion, acupoint massage, TCM exercise (e.g., Tai Chi), or combined TCM interventions.

*Comparators:* Conventional Western medical treatment, cognitive rehabilitation training, conventional Western medical treatment combined with rehabilitation training, or no intervention or blank control.

*Outcomes:* Cognitive function (e.g., Mini-Mental State Examination, MMSE; Montreal Cognitive Assessment, MoCA), activities of daily living (ADL), TCM syndrome scores, or adverse events.

*Study type:* Systematic reviews or meta-analyses of randomized controlled trials (RCTs).

### 2.4 Exclusion criteria

We excluded studies in which participants had cognitive impairment with other AIS-related secondary lesions or functional disorders (e.g., post-stroke pain, joint contracture, insomnia, depression, dysphagia), or cognitive impairment not caused by AIS (e.g., Alzheimer’s disease); ischemic stroke with other comorbid diseases; unclear diagnostic criteria; network meta-analyses; duplicate publications; studies with erroneous data or studies for which the full text was unavailable. Network meta-analyses were excluded because their methodological framework (indirect comparisons) differs substantially from standard systematic reviews. The AMSTAR 2 tool is not validated for network meta-analyses, and including them would introduce methodological heterogeneity that could compromise the validity of our evidence map.

### 2.5 Data extraction

Two authors (QW and QCL) used NoteExpress software to manage records and remove duplicates. Titles and abstracts were independently screened for relevance, followed by full-text screening of potentially eligible articles. Inter-rater agreement for full-text selection was high (kappa = 0.89). Disagreements regarding inclusion were resolved through discussion with a third reviewer (YQW). Extracted data included country or region, publication year, type of risk-of-bias assessment tool used, intervention types, sample size, and outcome measures.

### 2.6 Assessment of the methodological quality

Methodological quality was assessed using A Measurement Tool to Assess Systematic Reviews 2 (AMSTAR 2) (15). AMSTAR 2 is a practical tool for evaluating the quality of systematic reviews that include randomized and/or non-randomized studies of healthcare interventions. It consists of 16 items, of which items 2, 4, 7, 9, 11, 13, and 15 are critical domains. Each item was rated as “Yes,” “Partial Yes,” or “No”: (1) Yes, fully meets the criterion; (2) Partial Yes, partially meets the criterion; (3) No, does not meet the criterion. Overall methodological quality was categorized as high, moderate, low, or critically low based on item ratings: (1) High, no or one non-critical weakness; (2) Moderate, more than one non-critical weakness; (3) Low, one critical flaw with or without non-critical weaknesses; (4) Critically low, more than one critical flaw with or without non-critical weaknesses (15, 16). Two reviewers (QW and QL) independently conducted the assessments; discrepancies were discussed with a third reviewer (YW).

### 2.7 Presentation of the evidence map

First, characteristics of included SRs were described according to the PICO framework. Each study was classified and narratively summarized in tabular form. Based on AMSTAR 2 results, evidence bubble plots were designed generated to visualize the evidence. In these plots, each point represents one systematic review. The *x*-axis indicates the conclusion category (beneficial, probably beneficial, inconclusive, no differential effect, or harmful). The *y*-axis indicates the AMSTAR 2 quality rating (critically low, low, moderate, or high). Point size is proportional to the number of primary studies included in the review. Different colors represent distinct TCM intervention categories. The plots were created using R software (version 4.2.1; R Foundation for Statistical Computing, Vienna, Austria) with the ggplot2 package.

In addition, an evidence matrix was constructed to illustrate the distribution of systematic reviews across intervention categories and outcome measures. In this matrix, each bubble represents the number of systematic reviews reporting a given intervention—outcome pair. Bubble size is proportional to the review count, and bubble color indicates methodological quality according to AMSTAR 2 (red = critically low, blue = low). The matrix was also generated within the same R software environment.

## 3 Results

### 3.1 Literature search

**Figure 1:**
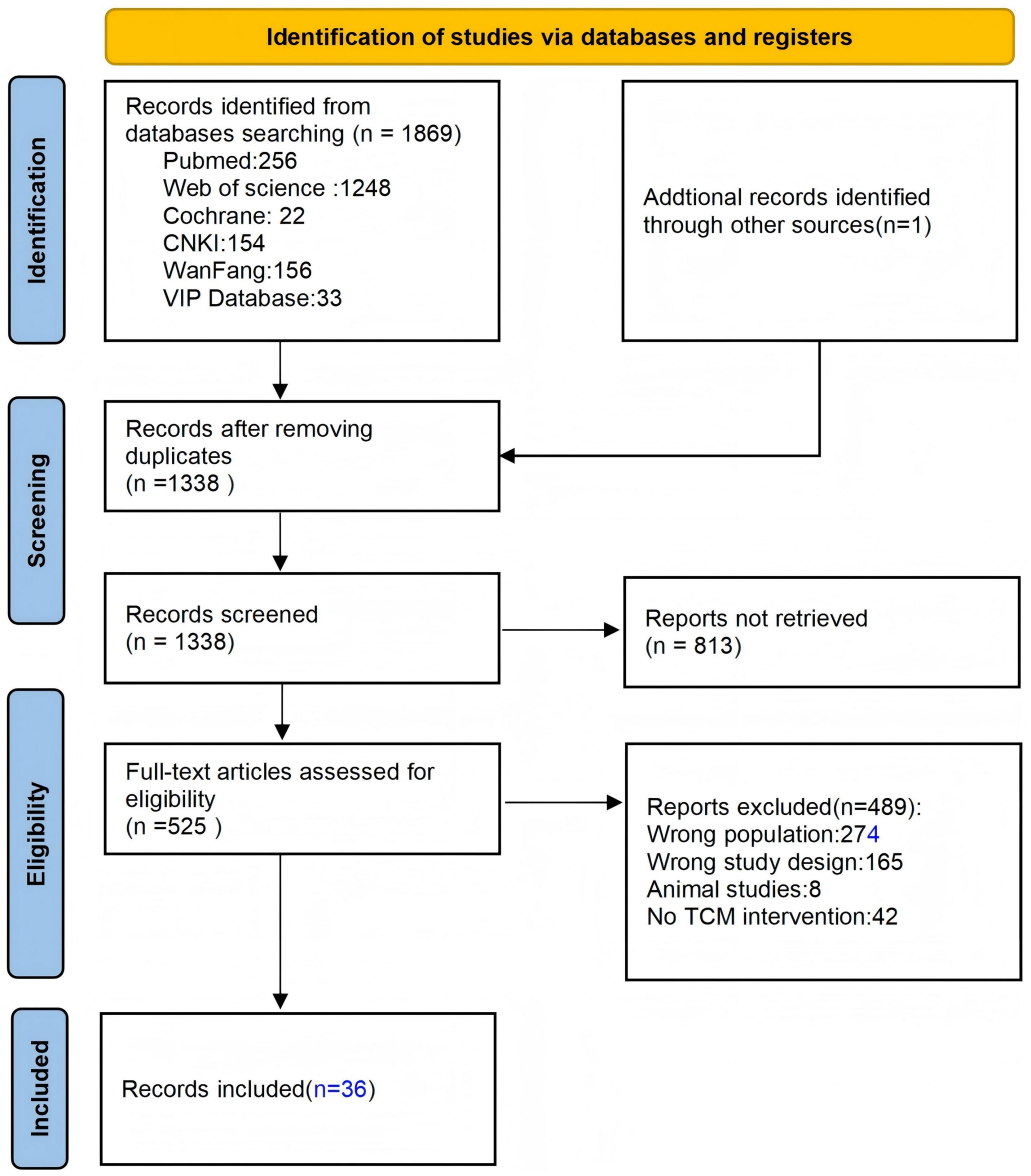
PRISMA flow diagram of the selection process.

A total of 1,869 records were retrieved from the databases. In addition, one record was identified through reference list screening. After deduplication, title and abstract screening, and full-text review, 36 systematic reviews were included for analysis. The study selection process is shown in Figure 1.

### 3.2 PICO characteristics of the included reviews

Across the included systematic reviews, the number of primary studies ranged from 7 to 39. Publication years spanned 2010–2024. The number of patients with PSCI included in each review ranged from 448 to 2,869, with ages ranging from 18 to 90 years. Regarding interventions, 27 reviews evaluated acupuncture, 4 assessed TCM herbal medicine, 3 examined moxibustion, 1 focused on auricular therapy, and 1 investigated TCM exercises (e.g., Baduanjin and Tai Chi). Control groups primarily involved three treatment approaches for PSCI: conventional Western medical treatment, cognitive rehabilitation training, or a combination of both. Intervention duration varied from 2 weeks to 6 months.

Outcomes were mainly evaluated across four domains: overall effectiveness, cognitive function, activities of daily living, and neurological function. Overall effectiveness was judged as “beneficial” in 33 reviews, “probably beneficial” in 1 review, and “inconclusive” in 2 reviews. All 36 reviews assessing cognitive function concluded that there was a “beneficial” effect. Among the 27 reviews evaluating activities of daily living, 26 reported “beneficial” findings, and 1 reported “no effect.” In addition, 5 reviews assessed neurological function, all of which reported “beneficial” effects of TCM interventions.

### 3.3 Assessment of methodological quality

**Figure 2:**
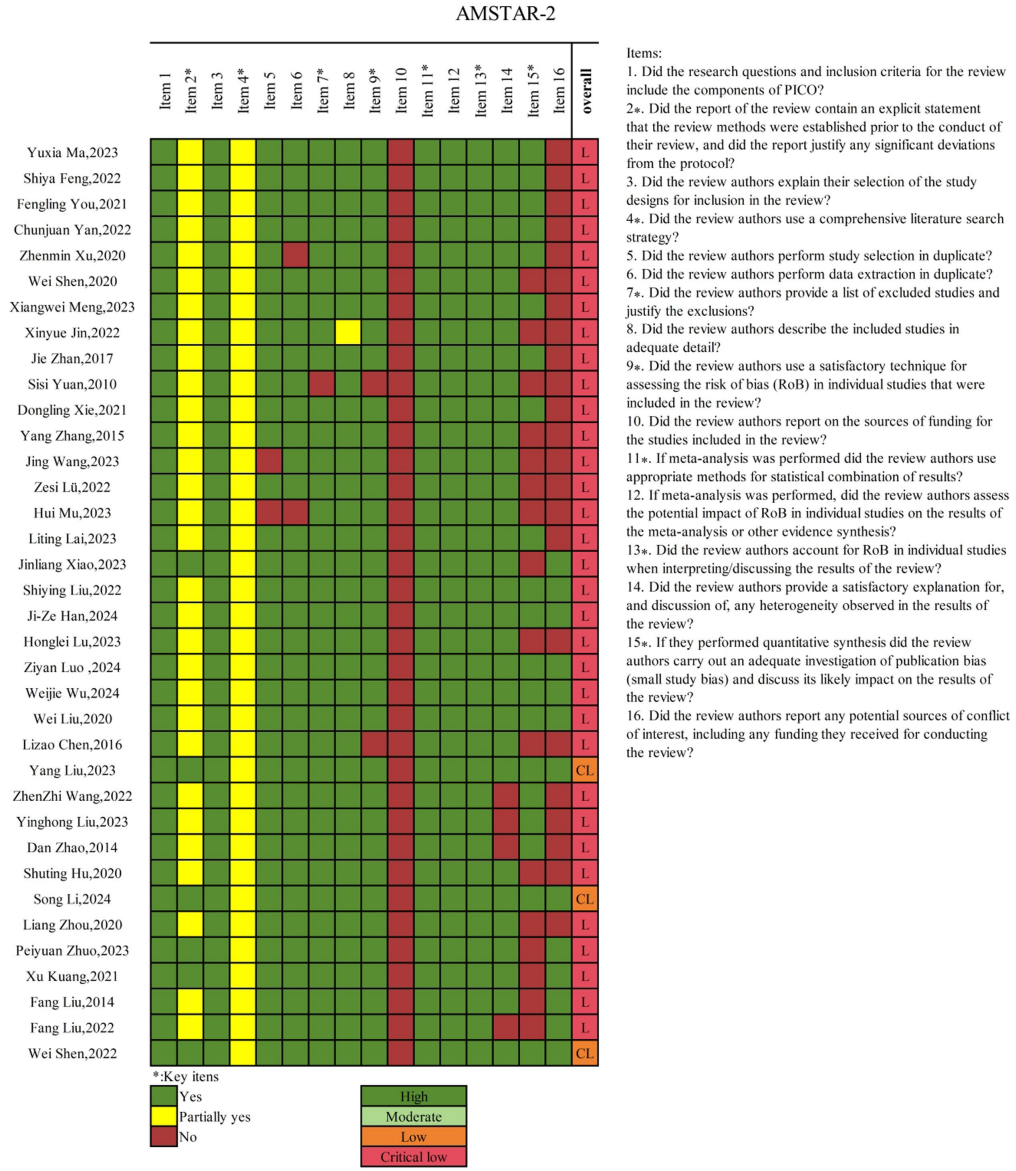
Methodological quality of the included systematic reviews.

According to the AMSTAR 2 assessment, 33 systematic reviews were rated as having “critically low” methodological quality (11, 12, 17–47), and 3 were rated as “low” quality (Figure 2) (8, 48, 49). The most frequent critical flaws were: (1) lack of prospective protocol registration (30/36, 83.3%); (2) insufficiently comprehensive literature searches (36/36, 100%); (3) absence of a list of excluded studies with reasons (35/36, 97.2%); and (4) failure to assess publication bias (16/36, 44.4%). The primary reasons for downgrading methodological quality included: (1) lack of prospective protocol registration or reporting of a predefined study plan in 30 reviews (11, 12, 17–47); (2) incomplete or insufficiently comprehensive literature search strategies in all 36 reviews (8, 11, 12, 17–47); (3) failure to describe the study selection process in 2 reviews (23, 43); (4) failure to describe the data extraction process in 2 reviews (23, 43); (5) absence of a list of excluded studies with reasons in 1 review (18); (6) inadequate reporting of basic characteristics of included studies in 1 review (46); (7) use of inappropriate tools for risk-of-bias assessment in 2 reviews (18, 31); (8) failure to report funding sources of the included primary studies in all 36 reviews (8, 11, 12, 17–49); (9) lack of explanation or discussion of heterogeneity in 4 reviews (32–34, 41); (10) failure to assess publication bias quantitatively in 16 reviews (18, 20–23, 25, 28, 31, 35, 37–41, 44, 46); and (11) lack of disclosure of potential conflicts of interest in 23 reviews (17–24, 27, 28, 31–37, 42–47).

### 3.4 Key findings from the evidence map of systematic reviews

**Figure 3:**
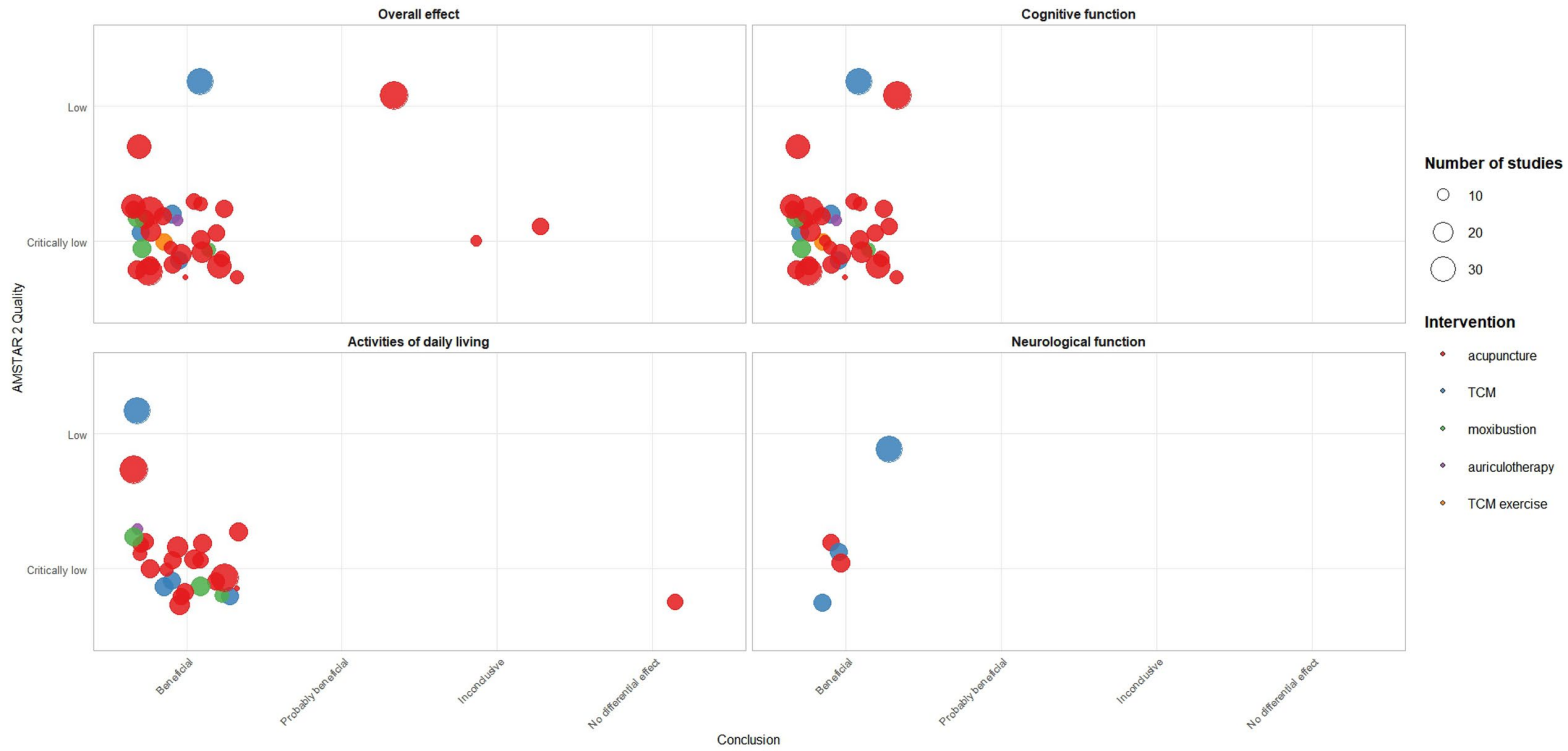
Evidence maps of TCM interventions for PSCI by outcome domain. Each point represents one systematic review. The *x*-axis indicates the conclusion (beneficial, probably beneficial, inconclusive, no differential effect), and the *y*-axis indicates AMSTAR 2 quality rating (critically low, low). Point size is proportional to the number of primary studies included in the review. Colors represent different TCM intervention categories.

*Overall effect*: As shown in Figure 3, 36 systematic reviews reported the overall effect. Of these, 33 reviews (91.7%) concluded that TCM interventions were beneficial for patients with PSCI, one review (2.8%) concluded probably beneficial, and two reviews (5.6%) judged the evidence as inconclusive. No reviews concluded “harmful” or “no effect.” The vast majority of reviews (34/36, 94.4%) were rated as critically low methodological quality (red bubbles for acupuncture, blue for herbal medicine). With only two reviews achieving low quality (blue bubbles for herbal medicine). Acupuncture (red) and herbal medicine (blue) contributed most of the evidence, while other modalities were sparsely represented. The predominance of red and blue bubbles with critically low quality ratings indicates that the favorable conclusions should be interpreted with caution.

*Cognitive function*: As shown in Figure 3, all 36 systematic reviews reported outcomes of cognitive function, and all found that TCM interventions were superior to comparators in improving cognitive function. Despite the consistently positive conclusions, 33 of 36 reviews (91.7%) were rated as critically low quality (red for acupuncture, blue for herbal medicine), with only three reviews achieving low quality (two red for acupuncture, one blue for herbal medicine). Acupuncture (red) dominated this domain, with substantial contributions also from TCM herbal medicine (blue). Evidence for moxibustion (green), auricular therapy (purple), and TCM exercises (yellow) was limited. The consistently positive conclusions across all 36 reviews are not supported by reliable methodological quality.

*Activities of daily living*: As shown in Figure 3, 27 systematic reviews reported outcomes related to activities of daily living. Among these, 26 reviews (96.3%) concluded that TCM interventions were superior to control conditions, while one review (3.7%) reported no differential effect. Methodological quality remained low overall: 25 reviews (92.6%) were critically low (red, blue, green, purple, yellow), and two reviews (7.4%) achieved low quality (red for acupuncture, one blue for herbal medicine). Acupuncture (red) provided the largest body of evidence, followed by TCM herbal medicine (blue) and moxibustion (green). Given that 92.6% of the contributing reviews were critically low quality, this apparent benefit should not be taken as definitive.

*Neurological function*: As shown in Figure 3, five reviews reported outcomes of neurological function, and all concluded that TCM interventions were superior to control conditions. Among these, four reviews were rated as critically low quality, and one review achieved low quality (blue bubble for herbal medicine). Evidence was contributed primarily by herbal medicine (blue) and acupuncture (red). With four reviews being critically low and only one low quality, no firm conclusion can be drawn about the true effect of TCM on neurological function.

### 3.5 Overall evidence matrix

**Figure 4:**
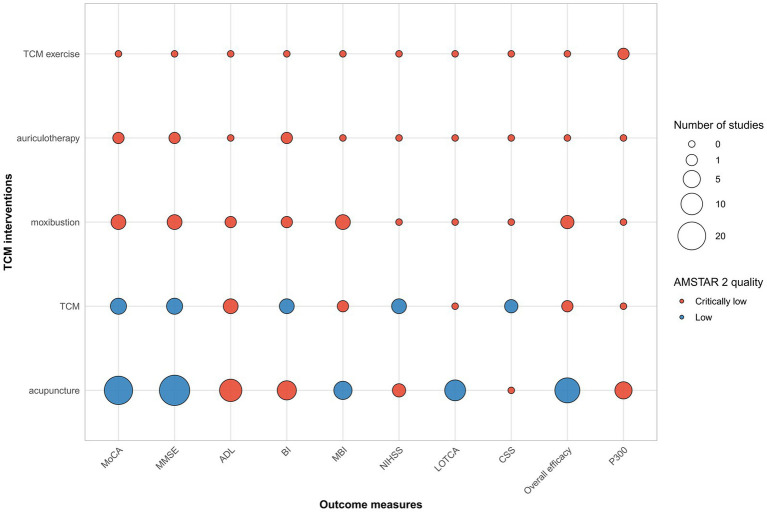
Evidence matrix of TCM interventions for PSCI. Bubble size is proportional to the number of systematic reviews for each intervention–outcome pair. Color represents AMSTAR 2 quality rating (red = critically low, blue = low). MoCA, Montreal Cognitive Assessment; MMSE, Mini-Mental State Examination; ADL, activities of daily living; BI, Barthel Index; MBI, Modified Barthel Index; NIHSS, National Institutes of Health Stroke Scale; LOTCA, Loewenstein Occupational Therapy Cognitive Assessment; CSS, Chinese Stroke Scale; Overall efficacy, overall effectiveness; P300, event-related potential P300.

Figure 4 presents a comprehensive overview of the evidence distribution across TCM intervention categories and outcome measures. Each bubble represents the number of systematic reviews reporting a given intervention-outcome pair, with bubble size proportional to the review count and color indicating the methodological quality according to AMSTAR 2 (red = critically low, blue = low).

Acupuncture was the most extensively studied intervention, with the largest bubbles concentrated in cognitive outcomes (MoCA and MMSE) and activities of daily living (ADL and BI/MBI). TCM herbal medicine also contributed substantial evidence, particularly for MoCA, MMSE, and ADL. In contrast, evidence for moxibustion, auricular therapy, and TCM exercises was limited across all outcome domains.

Strikingly, the vast majority of bubbles are red, visually underscoring that nearly all available evidence—regardless of intervention type or outcome—is of critically low methodological quality. A small number of blue bubbles appear for intervention–outcome pairs involving acupuncture (e.g., MoCA, MMSE, MBI, LOTCA, overall efficacy) and TCM (e.g., MoCA, MMSE, BI, NIHSS, CSS), reflecting the three systematic reviews rated as low quality.

## 4 Discussion

**Figure 5:**
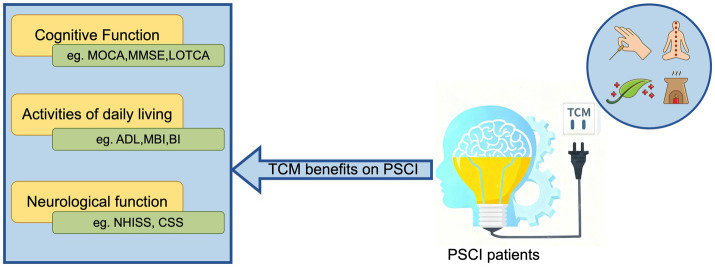
Graphical abstract of TCM interventions for post-stroke cognitive impairment. TCM, traditional Chinese medicine; PSCI, post-stroke cognitive impairment; MOCA, Montreal Cognitive Assessment; MMSE, Mini-Mental State Examination; Loewenstein Occupational Therapy Cognitive Assessment; ADL, activities of daily living; MBI, Modified Barthel Index; BI, Barthel Index; NIHSS, National Institutes of Health Stroke Scale; CSS, Chinese Stroke Scale. This figure was created with Figdraw, Export ID: UUSYT99f59.

In recent years, numerous clinical studies and systematic reviews have examined the effectiveness and safety of TCM for PSCI. To facilitate translation of existing evidence into practice, a prior overview of reviews on acupuncture for PSCI reassessed 18 systematic reviews and meta-analyses (50). Building on this work, the present study used evidence mapping to characterize the research landscape on TCM interventions for PSCI. Through a rigorous screening process, we ultimately included 36 systematic reviews or meta-analyses and integrated evidence across intervention types, outcomes, and methodological quality. This evidence map provides an intuitive overview of the field and highlights evidence gaps, thereby informing priorities for future research and optimization of clinical practice (Figure 5).

Our findings indicate that TCM interventions for PSCI are diverse. They mainly include acupuncture, herbal medicine, moxibustion, auricular stimulation, and TCM exercises (e.g., Baduanjin and Tai Chi). Acupuncture constituted the largest body of evidence (27 reviews), suggesting that it remains the most extensively studied TCM modality in PSCI. However, this should not be interpreted as evidence of superior efficacy. Rather, it may reflect research interest bias or publication bias toward acupuncture. The limited volume and poor quality of evidence for herbal medicine, moxibustion, and other TCM modalities preclude reliable comparative conclusions. Future head-to-head randomized controlled trials or network meta-analyses are needed to determine the relative effectiveness of different TCM interventions.

From an outcome perspective, included studies primarily evaluated four domains: overall effect, cognitive function, activities of daily living, and neurological function. All 36 reviews supported improvements in cognitive function (e.g., MMSE and MoCA), providing consistent evidence in favor of TCM interventions for core cognitive deficits. Among reviews assessing activities of daily living, the overwhelming majority (26/27) reported “beneficial” effects, and all reviews regarding neurological function similarly suggested benefit. Collectively, these findings imply that the effects of TCM interventions may be multidimensional, extending beyond improving cognitive function to enhancing functional independence and neurological function. This pattern contrasts with conventional management, which often focuses on risk-factor control and symptomatic pharmacotherapy with limited efficacy, thereby underscoring the potential complementary value of TCM in PSCI care. Recent literature has also highlighted the potential benefits of integrating TCM with conventional approaches in stroke management (51).

From a biomedical perspective, TCM interventions may influence multiple pathways relevant to post-stroke neurorehabilitation. Preclinical models show that acupuncture improves cerebral perfusion, attenuates neuroinflammation, and modulates energy metabolism. It also promotes synaptic plasticity and neurogenesis (52–54). Herbal formulas may exert antioxidant and anti-apoptotic effects, while moxibustion and auricular stimulation could influence autonomic regulation and cerebral blood flow (55, 56). These mechanisms align with contemporary understanding of post-stroke recovery, which emphasizes enhancing neuroplasticity and reducing secondary injury cascades.

In TCM theory, PSCI is traditionally attributed to deficiencies of qi and blood, or obstruction by phlegm and blood stasis (8). Although the theoretical frameworks differ, both perspectives converge on the notion that multisystem regulation—rather than a single molecular target—may be required for cognitive recovery after stroke. We present this brief TCM context to highlight the potential for future research that bridges traditional concepts with modern biomarkers.

However, intervention duration varied substantially (2 weeks to 6 months), which likely contributes to clinical and methodological heterogeneity. Given the complex disease trajectory of PSCI and differences in mechanisms across TCM modalities, optimal treatment duration remains unclear. Key unanswered questions include whether short-term benefits are durable, whether longer courses increase the risk of adverse events, and whether duration should be stratified by PSCI severity. These warrant focused comparative research.

Control conditions were also heterogeneous, including conventional Western medical treatment, cognitive rehabilitation, combined conventional Western medical treatment plus rehabilitation, and a blank control. Comparator selection directly affects effect estimation and interpretation. Usual care may include cholinesterase inhibitors or circulation-enhancing agents, both of which have limited efficacy and potential adverse effects. Comparisons against such regimens may accentuate the apparent advantages of TCM. Cognitive rehabilitation, a common non-pharmacological approach, may offer synergistic effects when combined with TCM. Yet, inadequate reporting of rehabilitation protocols (e.g., duration, frequency, content) can introduce baseline imbalance and compromise fairness in between-group comparisons. Blank control, while reflecting absolute effects more directly, may raise ethical concerns and is generally feasible only in milder cases. In addition, limited provision of background therapy may weaken clinical applicability. Such variability in comparators complicates the precise quantification of treatment effects and may hinder translation of evidence into practice.

Furthermore, the diagnostic criteria for PSCI varied across the included systematic reviews. Some used the MMSE threshold of <27, others used the MoCA <26, and still others relied on clinical judgment without a standardized cutoff. This diagnostic heterogeneity may contribute to variability in reported outcomes and should be addressed in future studies by adopting internationally harmonized diagnostic tools.

In addition, this study identified substantial shortcomings in the current literature on TCM interventions for PSCI, with the central limitation being generally poor methodological quality. Based on AMSTAR 2, of the 36 included reviews, 33 were rated as “critically low” and 3 as “low,” with none achieving a moderate or high rating. Accordingly, the overall certainty and trustworthiness of the evidence remain limited. This finding is consistent with earlier evidence maps in TCM stroke rehabilitation, where most systematic reviews were also of low or critically low quality (14, 57).

Outcome selection also leaves room for improvement. Commonly used scales (MMSE, MoCA, ADL) aid comparability with conventional research. However, outcomes capturing TCM-specific constructs (e.g., syndrome differentiation) are lacking. This may underestimate TCM’s benefits in constitutional regulation and symptom-pattern improvement. Moreover, outcome heterogeneity and inconsistent measurement tools reduce comparability across studies and may weaken pooled analyses. Evidence addressing long-term prognosis and recurrence risk remains limited, restricting the ability to inform comprehensive clinical decision-making (Table 1).

**Table 1:** Characteristics of included studies.

Author and year	Search date	Number of studies included	Participants	Age	Duration	Interventions	Comparisons	Quality assessment tool	Main outcomes	Conclusion
Yuxia Ma (2023) (17)	December 2023	15	918	46–65	NA	TCM exercises (e.g., Baduanjin, Tai Chi)	Conventional rehabilitation training or health education	Rob	⑩	Beneficial
Shiya Feng (2022) (27)	December 2022	18	1,334	NA	3w-4 m	Herbal medicine alone or combined with other TCM treatments	Western medication alone	Rob	①, ②, ③, ⑤, ⑨	Beneficial
Fengling You (2021) (36)	May 2021	16	1,283	57–72	2w-24w	TCM herbal formulas combined with Western medication	Western medication alone or blank control	Rob	①, ②, ③, ④, ⑥	Beneficial
Chunjuan Yan (2022) (42)	May 2022	20	1706	44–68	4w-12w	Acupuncture combined with cognitive training	Sham acupuncture with cognitive rehabilitation training, cognitive rehabilitation training alone, or medication	Rob	①, ②, ③, ⑨	Beneficial
Zhenmin Xu (2020) (43)	July 2020	15	1,301	NA	4w-12w	Acupuncture combined with cognitive training	Routine Western medication with or without other therapies	Rob	①, ②, ③, ④, ⑥	Inconclusive
Wei Shen (2020) (8)	December 2019	16	1,296	NA	3 m-6 m	Herbal medicine or Chinese patent medicine, with or without Western medication	Placebo or Western medication alone	Rob	①, ②, ③, ④, ⑥, ⑧,	Beneficial
Xiangwei Meng (2023) (45)	January 2022	14	993	52–65	20d-8w	Acupuncture combined with rTMS	Acupuncture only, rehabilitation therapy, cognitive therapy, rTMS alone, or basic treatment	Rob	①, ③, ⑤, ⑦	Beneficial
Xinyue Jin (2022) (46)	January 2022	12	824	NA	2w-12w	Moxibustion	Other treatments	Rob	①, ②, ⑤, ⑨,	Beneficial
Jie Zhan (2017) (47)	October 2016	14	896	NA	4w-8w	Electroacupuncture	Sham therapy, basic treatment, or other interventions	Rob	①, ②, ③, ⑨, ⑩	Beneficial
Sisi Yuan (2010) (18)	NA	9	620	NA	15d-180d	Acupuncture	Other effective non-acupuncture treatments	NA	②	Inconclusive
Dongling Xie (2021) (19)	May 2021	19	1,327	NA	3w-12w	Scalp acupuncture alone or combined with other therapies	Western medication alone or rehabilitation training	Rob	①, ②, ③, ④, ⑦, ⑨	Beneficial
Yang Zhang (2015) (20)	January 2015	11	789	30–75	4w-3 m	Acupuncture combined with cognitive training	Cognitive rehabilitation training alone or medication	Rob	②, ③, ⑨, ⑩	Beneficial
Jing Wang (2023) (21)	January 2021	15	555	NA	4w-3 m	Acupuncture, alone or combined with other treatments	Herbal medicine, Western drugs, or other treatments	Jadad	①, ②, ③, ⑨	Beneficial
Zesi Lü (2022) (22)	October 2021	7	507	NA	4w-6w	Warm needling combined with rehabilitation training and medication	Routine rehabilitation training and medication	Rob	①, ②, ④, ⑨	Beneficial
Hui Mu (2023) (23)	May 2022	16	1,278	NA	4w-12w	Acupuncture combined with cognitive training	Cognitive rehabilitation training, conventional treatment, or their combination	Rob	①, ②, ④, ⑨	Beneficial
Liting Lai (2023) (24)	April 2022	12	713	51–65	2w-2 m	Acupuncture combined with TMS (both high- and low-frequency) on the basis of conventional treatment	Conventional treatment and rehabilitation training	Rob	①, ②, ③	Beneficial
Jinliang Xiao (2023) (25)	August 2021	16	1,243	18–90	4w-12w	Scalp acupuncture combined with computer-assisted cognitive training	Conventional treatment	Rob	①, ⑤, ⑦	Beneficial
Shiying Liu (2022) (26)	April 2022	19	1,261	NA	2w-12w	Moxibustion, alone or partially combined with other therapies	Conventional treatment and rehabilitation training	Rob	①, ②, ④, ⑤	Beneficial
Ji-Ze Han (2024) (12)	January 2023	39	2073	53–68	12d-120d	Governor-vessel (Du-meridian) acupuncture	Various medications and rehabilitation therapies	Rob	①, ②, ③, ⑨	Beneficial
Honglei Lu (2023) (28)	November 2022	9	448	NA	4w-4 m	Auricular therapy, with or without other treatments	Conventional treatment and rehabilitation therapies (non-auricular therapies)	Rob	①, ②, ④	Beneficial
Ziyan Luo (2024) (11)	February 2024	29	2,169	51–71	2w-3 m	Acupuncture	Treatments other than acupuncture	Rob	①, ②, ⑦, ⑩	Beneficial
Weijie Wu (2024) (29)	May 2023	18	1,358	55–71	1 m-3 m	Acupuncture	Western medication alone	Rob	①, ②, ④, ⑨	Beneficial
Wei Liu (2020) (30)	January 2020	22	1856	52–68	3w-3 m	Acupuncture	Non-acupuncture treatments	Rob	①, ②, ④, ⑤	Beneficial
Lizao Chen (2016) (31)	November 2015	7	504	45–65	4w-10w	Scalp acupuncture alone or combined with other therapies	Rehabilitation training alone	NA	②, ⑨	Beneficial
Yang Liu (2023) (48)	May 2022	38	2,971	42–70	4w-12w	Acupuncture combined with cognitive training	Cognitive therapy alone	Rob	②, ⑤, ⑦	Probably beneficial
ZhenZhi Wang (2022) (32)	January 2021	14	2,402	48–68	4w-4 m	Acupuncture, alone or combined with other treatments	Conventional treatment	Rob	①, ②, ⑤, ⑦	Beneficial
Yinghong Liu (2023) (33)	December 2022	17	1,576	42–70	4w-12w	Scalp acupuncture combined with rehabilitation training	Other treatments	Jadad	①, ②, ③, ④, ⑥, ⑦, ⑨, ⑩	Beneficial
Dan Zhao (2014) (34)	NA	16	1,135	NA	28d-180d	Acupuncture therapy, alone or partially combined with other therapies	General treatment or other targeted therapies	Jadad	②, ③, ⑦, ⑨,	Beneficial
Shuting Hu (2020) (35)	October 2019	11	805	NA	4w-12w	Acupuncture at Baihui (GV20) and Shuigou (GV26)	Other therapies	Rob	①, ②, ⑨,	Beneficial
Song Li (2024) (24)	October 2024	28	1995	NA	3w-12w	Combined scalp acupuncture treatment	Single treatment	Rob	①, ②, ⑦, ⑨,	Beneficial
Liang Zhou (2020) (37)	December 2019	37	2,869	45–80	2w-12w	Acupuncture or electroacupuncture	Unclear	Rob	①, ②,	Beneficial
Peiyuan Zhuo (2023)(38)	July 2022	18	1,654	45–73	4w-3 m	Acupuncture combined with cognitive training	Cognitive training	Rob	①, ②, ⑤, ⑨,	Beneficial
Xu Kuang (2021) (39)	April 2019	28	2,144	45–71	4w-3 m	Acupuncture combined with other therapies	Standard treatment	Rob	①, ②,	Beneficial
Fang Liu (2014) (40)	March 2012	21	1,421	NA	2w-12w	Acupuncture combined with other therapies	Treatments other than acupuncture	Rob	②, ⑩	Beneficial
Fang Liu (2022) (41)	September 2021	17	1,290	NA	4w-12w	Moxibustion combined with other therapies	Conventional treatment and/or acupuncture, or medication	Rob	①, ②, ③, ⑤, ⑨,	Beneficial
Wei Shen (2022) (8)	February 2021	34	2,711	54–80	0.5 m-6 m	TCM combined with conventional Western medicine	Conventional Western medicine	Rob	①, ②, ④, ⑥, ⑧,	Beneficial

TCM, traditional Chinese medicine; rTMS, repetitive transcranial magnetic stimulation; Rob, Cochrane Risk of Bias Tool; Jadad, Jadad scale.

Outcomes: ① MOCA, Montreal Cognitive Assessment; ② MMSE, Mini-Mental State Examination; ③ ADL, activities of daily living, ④ BI, Barthel Index; ⑤ MBI, Modified Barthel Index; ⑥ NHISS, National Institutes of Health Stroke Scale; ⑦ LOTCA, Loewenstein Occupational Therapy Cognitive Assessment; ⑧ CSS, Chinese Stroke Scale; ⑨ overall efficacy, ⑩ P300 event-related potential.

The uniformly positive conclusions across all included reviews raise concerns about publication bias and selective reporting. Several factors may contribute: (i) all reviews originated from China, raising the possibility of cultural or regional publication bias favoring TCM interventions; (ii) small-study effects cannot be ruled out, as many included primary studies had small sample sizes; (iii) language restrictions (Chinese and English only) may have excluded negative results published in other languages. To mitigate these biases, future systematic reviews should adhere to PRISMA guidelines, register protocols prospectively, and include multilingual databases.

By applying evidence mapping, this study systematically integrated the current evidence. It visually clarifies the distribution of findings and key deficiencies. This helps to avoid redundant research and prioritize high-impact directions. Nevertheless, several limitations should be acknowledged.

First and most importantly, the methodological quality of the included systematic reviews was critically low in 91.7% of cases. This finding is concerning but not unexpected. It reflects systemic issues in the current evidence base for TCM interventions in PSCI. This has been noted in previous methodological studies (56). The prevalent critical flaws include: lack of protocol registration, insufficiently comprehensive searches, absence of excluded study lists, and failure to assess publication bias. These flaws mean that even consistently positive effect estimates should be interpreted with caution.

These deficiencies likely stem from several factors: (i) prospective registration was not widely practiced among Chinese researchers until recent years; (ii) space limitations in some Chinese-language journals may discourage exhaustive reporting; (iii) training in advanced systematic review methodology has only become widespread in China over the past decade. Importantly, these methodological weaknesses do not invalidate the consistent positive signals across outcomes. However, they substantially constrain the confidence that can be placed in them. The findings should therefore be considered hypothesis-generating rather than definitive. This underscores the urgent need for high-quality, prospectively registered systematic reviews and rigorous primary studies in this field. This is especially true for non-acupuncture modalities where evidence is both sparse and low quality.

Second, Conference abstracts and preprint servers were not searched. This may have resulted in missing preliminary or unpublished systematic reviews. However, we already included two dissertations. Most preprint systematic reviews are later published in peer-reviewed journals, we believe this omission has minimal impact on our conclusions.

Third, reporting standards for evidence mapping are not yet fully standardized. While our visualization approach highlights the key information, there remains scope to refine indicator selection and presentation formats.

Fourth, since we restricted eligible evidence to systematic reviews and meta-analyses. Primary studies were not included, limiting our ability to depict the full scope of research activity in this field.

## 5 Conclusion

In summary, existing systematic reviews suggest that TCM interventions may offer benefits for patients with PSCI across multiple outcome domains. However, given that over 90% of the included reviews were of critically low methodological quality, these findings should be interpreted as hypothesis-generating rather than definitive. The evidence map highlights substantial gaps and methodological weaknesses that must be addressed before firm clinical recommendations can be made. Future research should prioritize rigorous, prospectively registered systematic reviews and high-quality randomized controlled trials that adhere to established reporting standards, with particular attention to non-acupuncture modalities and harmonized outcome sets including TCM-relevant outcomes. Only through such efforts can a more reliable evidence base be established to support clinical decision-making and improve patient outcomes.

## Data Availability

The original contributions presented in the study are included in the article/supplementary material, further inquiries can be directed to the corresponding author.

## Footnotes

1
http://www.crd.york.ac.uk/prospero

2
http://www.clinicaltrials.gov

3
http://www.chictr.org.cn

## References

[1] Feske SK. Ischemic stroke. Am J Med. (2021) 134:1457–64. doi: 10.1016/j.amjmed.2021.07.027, 34454905

[2] Feigin VL Stark BA Johnson CO Roth GA Bisignano C Abady GG . Global, regional, and national burden of stroke and its risk factors, 1990–2019: a systematic analysis for the global burden of disease study 2019. Lancet Neurol. (2021) 20:795–820. doi: 10.1016/S1474-4422(21)00252-0, 34487721 PMC8443449

[3] Huang Y-Y Chen S-D Leng X-Y Kuo K Wang ZT Cui M . Post-stroke cognitive impairment: epidemiology, risk factors, and management. J Alzheimer's Dis. (2022) 86:983–99. doi: 10.3233/JAD-215644, 35147548

[4] Li Y Cui R Liu S Qin Z Sun W Cheng Y . The efficacy and safety of post-stroke cognitive impairment therapies: an umbrella review. Front Pharmacol. (2023) 14:1207075. doi: 10.3389/fphar.2023.1207075, 37693907 PMC10483224

[5] Birks JS Harvey RJ. Donepezil for dementia due to Alzheimer's disease. Cochrane Database Syst Rev. (2018) 2018:6. doi: 10.1002/14651858.CD001190.pub3, 29923184 PMC6513124

[6] Gittler M Davis AM. Guidelines for adult stroke rehabilitation and recovery. JAMA. (2018) 319:820–1. doi: 10.1001/jama.2017.22036, 29486016

[7] Kreiger K Weiss E Fluri F. Novel therapies for post-stroke cognitive impairment: a systematic review. Front Neurol. (2025) 16, 01–15. doi: 10.3389/fneur.2025.1569329, 40496133 PMC12150855

[8] Shen W Fan X Wang L Zhang Y. Traditional Chinese medicine for post-stroke cognitive impairment: a systematic review and meta-analysis. Front Pharmacol. (2022) 13:816333. doi: 10.3389/fphar.2022.816333, 35237166 PMC8883343

[9] Ning B Zhu X Wu X Zhu W Wang R Qi C . Efficacy of different traditional Chinese medicine decoctions in the treatment of ischemic stroke: a network meta-analysis. Front Pharmacol. (2024) 15:1486458. doi: 10.3389/fphar.2024.1486458, 39555103 PMC11565597

[10] Sowmiya S Begum RF Dhivya L Rajendran P Harikrishnan N Singh SA. Traditional, complementary, and integrative medicine in the management of ischemic stroke: a narrative review. Front Pharmacol. (2025) 16:1561688. doi: 10.3389/fphar.2025.1561688, 40520194 PMC12163044

[11] Luo Z Li W Jiang J Sun J Zhang M Zhang Y . Effect of acupuncture on cognitive function in patients with post-stroke cognitive impairment: a systematic review and Meta-analysis. Brain Behav. (2024) 14:e70075. doi: 10.1002/brb3.70075, 39402813 PMC11473547

[12] Han J-Z Yang Y Wang Y-F Feng JH Song CN Wu WJ . Effectiveness and safety of governor vessel acupuncture therapy for post-stroke cognitive impairment: a meta-analysis of randomized controlled trials. Ageing Res Rev. (2024) 99:102355. doi: 10.1016/j.arr.2024.102355, 38942201

[13] Hetrick SE Parker AG Callahan P Purcell R. Evidence mapping: illustrating an emerging methodology to improve evidence-based practice in youth mental health. J Eval Clin Pract. (2010) 16:1025–30. doi: 10.1111/j.1365-2753.2008.01112.x, 20337833

[14] Choi T Jun JH Lee HW Yun J Joo MC Lee MS . Traditional Chinese medicine interventions in the rehabilitation of cognitive and motor function in patients with stroke: an overview and evidence map. Front Neurol. (2022) 13:13. doi: 10.3389/fneur.2022.885095, 35655620 PMC9152210

[15] Shea BJ Reeves BC Wells G Thuku M Hamel C Moran J . AMSTAR 2: a critical appraisal tool for systematic reviews that include randomised or non-randomised studies of healthcare interventions, or both. BMJ. (2017):j4008. doi: 10.1136/bmj.j4008, 28935701 PMC5833365

[16] Pieper D Lorenz RC Rombey T Jacobs A Rissling O Freitag S . Authors should clearly report how they derived the overall rating when applying AMSTAR 2—a cross-sectional study. J Clin Epidemiol. (2021) 129:97–103. doi: 10.1016/j.jclinepi.2020.09.046, 33049325

[17] Ma YX Yuan Y Yang YY . Meta-analysis of traditional Chinese exercises in patients with poststroke cognitive impairment. *Huli Guanli Zazhi* (J Nurs Manage). (2024) 24:266–71. [in Chinese]

[18] Yuan SS Zhang SY. Meta-analysis of acupuncture for poststroke cognitive impairment. *Zhongguo Minzu Minjian Yiyao* (Chin J Ethnic Med). (2010) 19:47–8. doi: 10.3969/j.issn.1007-8517.2010.09.031 [in Chinese]

[19] Xie DL Yang K Xie HH Cong DY. Meta-analysis of the clinical efficacy of scalp acupuncture for poststroke cognitive impairment. *Zhongyiyao Daobao* (Tradit Chin Med Herald). (2021) 27:130–6.

[20] Zhang Y Tang W Song XG Wu S Zhang GY Xu H. Systematic review and meta-analysis of acupuncture combined with cognitive rehabilitation training for poststroke cognitive impairment. *Shanghai Zhenjiu Zazhi* (Shanghai J Acupuncture Moxibustion). (2015) 34:1013–20. doi: 10.13460/j.issn.1005-0957.2015.10.1013 [in Chinese]

[21] Wang J. Systematic review of acupuncture for poststroke cognitive impairment. *Zhongyi Linchuang Yanjiu* (Clin J Chin Med). (2023) 15:114–20. doi: 10.3969/j.issn.1674-7860.2023.04.022 [in Chinese]

[22] Lv ZC Lin SH Lin N Guo JY Liu F. Meta-analysis of the rehabilitation effects of warm acupuncture/moxibustion on poststroke cognitive dysfunction. *Mudanjiang Yixueyuan Xuebao* (J Mudanjiang Med Univ). (2022) 43:96–101. [in Chinese]

[23] Mu H Tang JQ Huang HL Zu GX. Systematic review of acupuncture combined with cognitive training for poststroke cognitive impairment. *Shenjing Sunshang Gongneng Chongjian* (Neural Injury Funct Reconstr). (2023) 7:373–9.doi: 10.16780/j.cnki.sjssgncj.20220643 [in Chinese]

[24] Lai LT Lin R Peng ZH Xu MZ Tang CZ Cui SY. Systematic review and meta-analysis of acupuncture combined with repetitive transcranial magnetic stimulation for poststroke cognitive impairment. *Guangzhou Zhongyiyao Daxue Xuebao* (J Guangzhou Univ Chin Med). (2023) 40:1838–46. [in Chinese]

[25] Xiao J Wang T Ye B Tang C. Scalp acupuncture and computer assisted cognitive rehabilitation for stroke: a meta-analysis of randomised controlled trials. Heliyon. (2023) 9:e18157. doi: 10.1016/j.heliyon.2023.e18157, 37501979 PMC10368847

[26] Liu SY Guo W Wang JP Wu BY. Systematic review of the effects of moxibustion on rehabilitation in patients with poststroke cognitive impairment. *Xunzheng Huli* (Evidence-Based Nursing). (2022) 8:3138–44. doi: 10.12102/j.issn.2095-8668.2022.23.003 [in Chinese]

[27] Feng SY. *Systematic evaluation of the clinical efficacy of chinese herbal medicine for poststroke cognitive impairment*. (master's thesis). Hubei University of Chinese Medicine (2023)

[28] Lu HL Zhuang LL Zhang R. Meta-analysis of auricular therapy for poststroke cognitive dysfunction. *Xunzheng Huli* (Evidence-Based Nursing). (2023) 9:1345–9. doi: 10.12102/j.issn.2095-8668.2023.08.003 [in Chinese]

[29] Wu W Song C Yang Y Hu Y Lin H. Acupuncture for cognitive impairment after stroke: a systematic review and meta-analysis. Heliyon. (2024) 10:e30522. doi: 10.1016/j.heliyon.2024.e30522, 38765166 PMC11098789

[30] Liu W Rao C Du Y Zhang L Yang J. The effectiveness and safety of manual acupuncture therapy in patients with Poststroke cognitive impairment: a Meta-analysis. Neural Plast. (2020) 2020:1–15. doi: 10.1155/2020/8890521

[31] Chen LZ Li W Wang JQ . Meta-analysis of scalp acupuncture for poststroke cognitive impairment. *Zhongyiyao Daobao* (Trad Chin Med Herald). (2016) 22:84–7. [in Chinese]

[32] Wang Z-Z Sun Z Zhang M-L Xiong K Zhou F. Systematic review and meta-analysis of acupuncture in the treatment of cognitive impairment after stroke. Medicine. (2022) 101:e30461. doi: 10.1097/MD.0000000000030461, 36254056 PMC9575739

[33] Liu YH. *Meta-analysis of scalp acupuncture combined with rehabilitation training for poststroke cognitive dysfunction*. (master's thesis). Wuhan: Hubei University of Chinese Medicine (2023) [in Chinese].

[34] Zhao D Li ZW Li P. Systematic review of different acupuncture methods for poststroke cognitive impairment. *Sichuan Zhongyi* (Sichuan J Trad Chin Med). (2014) 32:155–8.

[35] Hu ST Pi M. Systematic review and meta-analysis of acupuncture at Baihui (GV20) and Shuigou (GV26) for poststroke cognitive impairment. *Guangzhou Zhongyiyao Daxue Xuebao* (J Guangzhou Univ Chin Med). (2020) 37:2035–42. doi: 10.13359/j.cnki.gzxbtcm.2020.10.037

[36] You FL Xia GF Liu YL Cai J. Meta-analysis of Chinese herbal compound prescriptions combined with Western medicine for poststroke cognitive impairment. *Zhongxi Yijiehe Xinnao Xueguanbing Zazhi* (Chin J Integr Med Cardio-Cerebrovas Dis). (2023) 21:4091–100.

[37] Zhou L Wang Y Qiao J Wang QM Luo X. Acupuncture for improving cognitive impairment after stroke: a meta-analysis of randomized controlled trials. Front Psychol. (2020) 11:549265. doi: 10.3389/fpsyg.2020.549265, 33424671 PMC7793937

[38] Zhuo P Huang L Lin M Chen J Dai Y Yang M . Efficacy and safety of acupuncture combined with rehabilitation training for poststroke cognitive impairment: a systematic review and meta-analysis. J Stroke Cerebrovasc Dis. (2023) 32:107231. doi: 10.1016/j.jstrokecerebrovasdis.2023.107231, 37473532

[39] Kuang X Fan W Hu J Wu L Yi W Lu L . Acupuncture for post-stroke cognitive impairment: a systematic review and meta-analysis. Acupunct Med. (2021) 39:577–88. doi: 10.1177/09645284211009542, 34074151

[40] Liu F Li Z-M Jiang Y-J Chen L-D. A meta-analysis of acupuncture use in the treatment of cognitive impairment after stroke. J Altern Complement Med. (2014) 20:535–44. doi: 10.1089/acm.2013.0364, 24915606 PMC4086349

[41] Liu F Lyu Z Lin S Li Z Xiu H. Effects of moxibustion on cognition and activities of daily living in post-stroke cognitive impairment: a systematic review and meta-analysis of randomized controlled trials. J Nurs Scholarsh. (2023) 55:464–76. doi: 10.1111/jnu.12846, 36345735

[42] Yan CJ Ding FF Guo P . Meta-analysis of acupuncture combined with cognitive training for poststroke cognitive impairment. *Yatai Chuantong Yiyao* (Asia-Pacific Trad Med). (2023) 19:140. [in Chinese]

[43] Xu ZM Liao X Jia M . Systematic review of the effectiveness and safety of acupuncture for poststroke cognitive impairment. *Beijing Zhongyiyao* (Beijing J Trad Chin Med). (2023) 39:1117–22. doi: 10.16025/j.1674-1307.2020.11.001 [in Chinese]

[44] Shen W Zeng ZX Jin XL . Systematic review of the efficacy and safety of Chinese herbal medicine for poststroke cognitive impairment. *Zhongguo Shiyan Fangjixue Zazhi* (Chin J Exp Tradit Med Formulae). (2020) 26:185–93. doi: 10.13422/j.cnki.syfjx.20201151 [in Chinese]

[45] Meng XW Ren YY Dang S Zhao BY Zhang XL. Meta-analysis of acupuncture combined with repetitive transcranial magnetic stimulation for poststroke cognitive impairment. *Zhongxi Yijiehe Xinnao Xueguanbing Zazhi* (Chin J Integr Med Cardio-Cerebrovas Dis). (2023) 21:1941–8. [in Chinese]

[46] Jin XY Huang JH You XF. Meta-analysis of moxibustion for poststroke cognitive impairment. *Zhongguo Yiyao Kexue* (China Med Pharm). (2022) 12 doi: 10.3969/j.issn.2095-0616.2022.19.020 [in Chinese]

[47] Zhan J Wang X Cheng N Tan F. Electroacupuncture for post-stroke cognitive impairment: a systematic review and meta-analyses. *Zhongguo zhen jiu* (Chin Acupuncture Acupuncture). (2017) 37:1119–25. doi: 10.13703/j.0255-2930.2017.10.025, 29354984

[48] Liu Y Chen F Qin P Zhao L Li X Han J . Acupuncture treatment vs. cognitive rehabilitation for post-stroke cognitive impairment: a systematic review and meta-analysis of randomized controlled trials. Front Neurol. (2023) 14:1035125. doi: 10.3389/fneur.2023.1035125, 36846126 PMC9946978

[49] Li S Dai A Zhou Y Chen X Chen Y Zhou L . Efficacy of combination scalp acupuncture for post-stroke cognitive impairment: a systematic review and meta-analysis. Front Neurosci. (2024) 18:1468331. doi: 10.3389/fnins.2024.1468331, 39678533 PMC11638195

[50] Xia MZ Yin ZH Chen ZH . An overview of systematic reviews on acupuncture interventions for poststroke cognitive impairment. Shijie Kexue Jishu–Zhongyiyao Xiandaihua (World Sci Technol/Modern Trad Chin Med Mater Med). (2023) 25:2821–31. [in Chinese]

[51] Liu X Qian Z Li Y Wang Y Zhang Y Zhang Y . Unveiling synergies: integrating TCM herbal medicine and acupuncture with conventional approaches in stroke management. Neuroscience. (2025) 567:109–22. doi: 10.1016/j.neuroscience.2024.12.043, 39730019

[52] Pan P Ma Z Zhang Z Ling Z Wang Y Liu Q . Acupuncture can regulate the peripheral immune cell spectrum and inflammatory environment of the vascular dementia rat, and improve the cognitive dysfunction of the rats. Front Aging Neurosci. (2021) 13:706834. doi: 10.3389/fnagi.2021.706834, 34349636 PMC8328226

[53] Bu Y Li W-s Lin J . Electroacupuncture attenuates immune-inflammatory response in hippocampus of rats with vascular dementia by inhibiting TLR4/MyD88 signaling pathway. Chin J Integr Med. (2022) 28:153–61. doi: 10.1007/s11655-021-3350-5, 34913150 PMC8672855

[54] Zhou X Cui G Tseng HHL Lee SMY Leung GPH Chan SW . Vascular contributions to cognitive impairment and treatments with traditional Chinese medicine. Evid Based Complement Alternat Med. (2016) 2016:9627258. doi: 10.1155/2016/9627258, 28042305 PMC5141557

[55] Zhang X Wu B Nie K Jia Y Yu J. Effects of acupuncture on declined cerebral blood flow, impaired mitochondrial respiratory function and oxidative stress in multi-infarct dementia rats. Neurochem Int. (2014) 65:23–9. doi: 10.1016/j.neuint.2013.12.004, 24361538

[56] Li N Wang H Liu H Zhu L Lyu Z Qiu J . The effects and mechanisms of acupuncture for post-stroke cognitive impairment: progress and prospects. Front Neurosci. (2023) 17:1211044. doi: 10.3389/fnins.2023.1211044, 37397457 PMC10309044

[57] Jiang Y Zhong CC Wang BH Xu SS Ho FF Kwong MH . Methodological quality of systematic reviews on orally administered Chinese herbal medicine published in Chinese between 2021 and 2022: a cross-sectional study. J Integr Med. (2025) 23:492–501. doi: 10.1016/j.joim.2025.07.005, 40858491

